# Morphological Mutations: Lessons from the Cockscomb

**DOI:** 10.1371/journal.pgen.1004979

**Published:** 2015-03-19

**Authors:** Denis Headon

**Affiliations:** The Roslin Institute and Royal (Dick) School of Veterinary Studies, University of Edinburgh, Roslin, United Kingdom; University of Bern, Switzerland

Enormously diverse anatomies have arisen through the course of animal evolution. These varied morphologies are encoded within different genomes, interpreted and implemented through the process of development. Genes controlling anatomical development tend to be highly pleiotropic, operating within large networks to guide the formation of many functionally unrelated structures. This property, together with the high degree of sequence conservation of protein coding sequences between species, has led to the suggestion that evolution of form is driven mostly by mutations in the noncoding parts of the genome responsible for regulation of gene expression. Mutation of gene regulatory sequences could, in principle, influence gene activity in only one or a few tissues, enabling new morphologies with beneficial effects in one organ to be selected while avoiding potentially harmful effects to other structures that might arise from coding sequence mutation [1].

Systematic analyses of genomic regions under selection have supported the view that noncoding mutations play a greater role in adaptation to new environments than coding sequence mutations [2]. However, such population-level, genome-wide studies do not distinguish between morphological and other types of adaptive variation and do not identify the specific mutations associated with distinct traits. A number of phenotype-led studies in which the genetic basis of specific traits has been identified also support the view that morphological evolution often occurs through *cis*-regulatory mutation [3]. A problem with these trait-directed studies is that they have been done in diverse organisms and have focused on diverse traits, yielding disparate examples that make it difficult to draw general conclusions. In addition, the overwhelming majority of protein-coding mutations with which geneticists are familiar impair, rather than enhance, gene function, and it is not yet clear how this lesson might translate to non-coding mutations. A set of studies published over the past five years by Andersson and colleagues addresses these issues by focussing on the genetic basis for multiple variant forms of a single structure. These enable a test of the “regulatory hypothesis” without bias, and, further, address whether there is any particular form taken by the underlying mutations themselves. The molecular basis of duplex-comb, reported in this issue of *PLOS Genetics*, completes this informative collection of morphological mutations [4].

**Fig 1:**
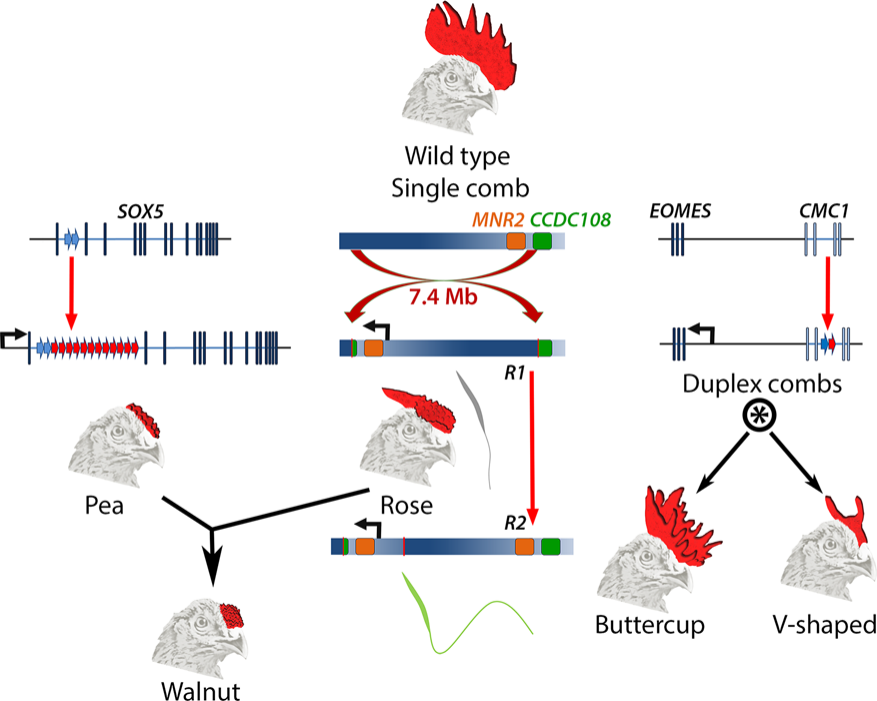
Major comb variants and their genetic basis. The wild type chicken comb is a single serrated blade. Pea-comb is caused by expansion of an existing tandem duplication in *SOX5*, leading to ectopic expression of this gene in the mesenchyme of the embryonic comb. Rose-comb is caused by a large inversion that triggers ectopic expression of *MNR2* in the same cells. Rose-comb allele *R1* includes a disrupted *CCDC108* gene and is associated with poor sperm quality. A second Rose-comb allele, *R2*, which is derived from *R1*, has an intact *CCDC108* gene and restored fertility. The Walnut-comb variant is caused by epistatic interaction between Pea- and Rose-comb alleles. Duplex-comb phenotypes are caused by formation of a new tandem duplication within *CMC1*, resulting in ectopic expression of the nearby gene *EOMES* in the ectoderm of the embryonic comb-forming region. Two duplex-comb forms exist, which are distinguished by a second mutation or a linked modifier allele (indicated by *). Genetic loci are not drawn to scale.

The chicken’s comb, which in the wild type is a single serrated blade on top of the head, has taken on a range of new shapes in domestic chickens (Fig. 1). These alternate comb forms have long been known to have a simple heritable basis, serving as major traits for the earliest demonstrations of Mendelian inheritance in animals [5] and yielding a first example of epistatic interaction between genes [6]. Andersson and colleagues set out to identify the genetic basis for the three major variants of the comb, Pea-comb (a smaller comb typically composed of three knobbed ridges), Rose-comb (flattened with papillae towards the beak and tapered to the back), and duplex-combs (either a full comb duplication or small paired horns), providing a systematic set in which to discern whether any particular type of mutation drives alteration of form. The commonalities uncovered are striking.

All three comb variants are underlain by regulatory mutations that are structural, rather than single nucleotide changes, and each causes ectopic expression of a transcription factor. Pea-comb is caused by an approximately 30-fold expansion of a pre-existing tandem duplication in noncoding sequence at *SOX5* [7]; Rose-comb by a large inversion, which induces expression of *MNR2* [8]; and the duplex-comb phenotypes are now revealed to be a result of a tandem duplication in an intron of *CMC1*, which triggers expression of the neighbouring gene *EOMES* [4]. These acquisitions of new expression domains at the prospective comb region occur despite there being very little new sequence generated by the mutations, the bulk of which constitute amplification or rearrangement of existing sequences. It will be interesting to determine the gene regulatory mechanisms underlying the effect of the tandem expansions; whether these disrupt endogenous repressive elements, cause de novo formation of site-specific enhancers, or exert a more general locus-wide effect to permit the action of previously cryptic enhancers. The resulting ectopic expression of each transcription factor presumably amplifies the effect of the causative mutation into altered expression of many genes, thereby modifying the intercellular signalling that controls comb outgrowth [9].

Variation in domestic animals has been used as a guide to understand variation between species since the beginning of evolutionary thinking. However, sheltered from the full force of natural selection by human management, domesticated populations may be able to harbour crude mutations that would not be maintained in wild populations. Here, too, these comb studies have lessons, showing that further evolution of the original mutant alleles occurs either to reduce pleiotropic effects on fitness or to achieve finer tuning of the selected morphological phenotype. The former phenomenon is exemplified by Rose-comb, the original mutant allele of which causes sub-fertility, which has acquired a second rearrangement to repair this defect [8]. Further refinement of form is illuminated by the duplex-comb’s two distinct shapes, which carry the same driving mutation, indicating that a second mutation arising at this site, or possibly a closely linked modifier allele, determines the difference between these morphologies [4,10]. Taken together, this catalogue of mutations hints at the types of genomic change that tend to serve as the source of morphological variation within, and perhaps between, animal species.
